# A case of asymmetrical monocephalus dipygus (*tetrapus dibrachius*) in a male Holstein calf in Iran

**Published:** 2016-06-15

**Authors:** Behrokh Marzban Abbasabadi, Aliakbar Ahmadzadeh, Shahab Ramezanpour, Amir Reza Hajati Ziabari

**Affiliations:** 1 *Department of Basic Veterinary Sciences, Faculty of Veterinary Medicine, Amol Universityof Special Modern Technologies, Amol, Iran;*; 2 *DVM Student, Faculty of Veterinary Medicine, Islamic Azad University, Babol Branch, Babol, Iran;*; 3 *Department of Pathobiology, Faculty of Veterinary Medicine, University of Tehran, Tehran, Iran; *; 4 *Young Researchers and Elite Club, Faculty of Veterinary Medicine, Islamic Azad University, Babol branch, Babol, Iran.*

**Keywords:** Conjoined twins, Holstein calf, Monocephalus dipygus, *Tetrapus dibrachius*

## Abstract

Dipygus is a teratological fetus with a double pelvis, genitals, and extremities. Congenital duplications in cattle are rare. Caudal duplication is more common in sheep and pigs while cranial duplications seem to be predominant in cattle. Asymmetric or parasitic conjoined twins consisting of an incomplete twin (parasite) attached to the body of a fully-developed twin (autosite). This report deals with a male Holstein calf with two extra limbs, in the pelvic region which were directed ventrally between the two normal hind limbs. The extra limbs were completely developed in one side and in other side just a bony mass were observed. So classification has been made as asymmetrical attached twins. The genital system was not affected and just one extra kidney-like structure was found. To the authors’ best knowledge, this is the first report of asymmetrical monocephalus dipygus (*tetrapus dibrachius*) in a male Holstein calf in Iran.

## Introduction

Dipygus or caudal duplication is a rare anomaly in the calf that is structurally related to monozygotic twins.^1^ According to the extension of the anomaly, duplicated cases are classified as monocephalus tripus dibrachius, monocephalus tetrapus dibrachius and cephalo-theracopagus.^2^ Definitive etiological information and data about embryo duplications are limited.^3^ It is assumed to be caused by genetic or environmental factors, or by their interaction or by ageing ova.^4^^-^^6^

To the author’s best knowledge just one case of symmetrical monocephalus dipygus (*tetrapus dibrachius*) with urogenital defects in a female Holstein calf has been reported in Kerman, south of Iran^1^, So this is the first report of asymmetrical monocephalus dipygus (*tetrapus dibrachius*) in a male Holstein calf in Iran.

## Case Description

A dead male Holstein calf with two extra pelvic limbs was brought to a private clinic in August 2015 in Babol, northern Iran, and was later sent to the veterinary teaching hospital of Islamic Azad University of Babol, Iran. 

**Fig. 1:**
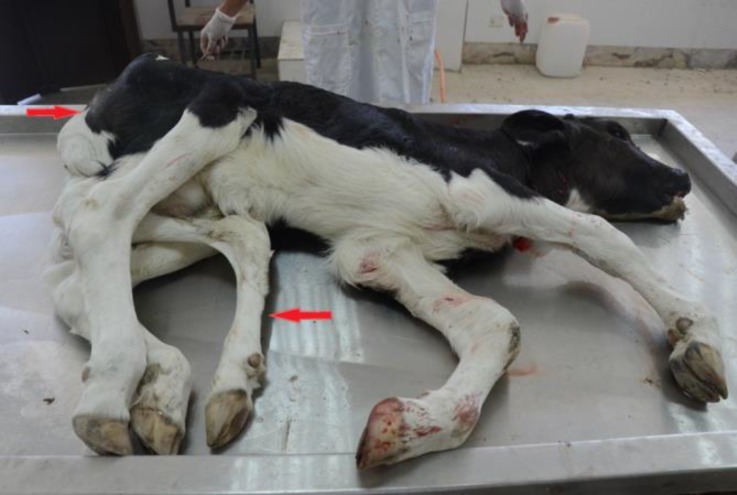
Extra limbs in the affected Holstein calf.

The gross external features from the head down to the perineum and pelvis were essentially normal. Within the region of the pelvic and perineum, there were two extra limbs, which were directed ventrally between the two normal hind limbs (Fig. 1).

**Fig. 2.**
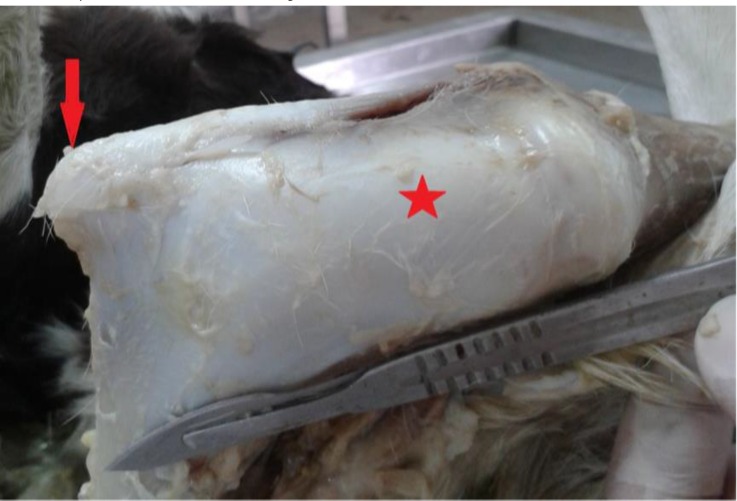
Patella (arrow) was just like a prominence proximal to the abnormal distal end of femur (star

The internal examination started from the pelvic region. The pelvic symphysis was not formed, and the two hip bones were separated. The hip bones were smaller than the normal size. On the left side, and at the medial border of the pubis and ischium, an articular surface was made, and the head of the extra femur was attached there by a ligament like the round ligament. The length of the extra femur was normal but because of the insufficient growth of muscular mass the diameter of femoral region was not that much. The head and the greater trochanter of the extra femur were abnormal, and the lesser trochanter was absent. The patella was not seen as a separate bone and was just like a prominence proximal to the abnormal distal end of femur (Fig. 2). Among the patellar ligaments, just the middle patellar ligament was developed. Among the ligaments between femur and tibia, only the cranial and caudal cruciate ligaments were seen, and the two bones were connected by a broad connective tissue. The knee joint was flexed, and the extra limb was thus above the ground. The ankylosis was also observed in other joints of extra limbs. Fibula was absent but tibia and the other bones of hind paw had the same length as the normal limbs. Musculature of extra limb was significantly reduced or absent, which could be a reason for arthrogryposis. The knee and tarsal joints were fixed in flexed position, and the fetlock, pastern, and coffin joints were fixed in extended position. The flexion angels of the knee and digits were cranially, and the flexion angle of the tarsal joint was caudally. 

An articular surface like the one at the left side was observed on the right hip bone; however, it was smaller, and just an approximately 20 cm long bony structure was attached to it. 

At necropsy, a parenchymal mass similar to an abnormal kidney was observed in sublumbar region but it did not contain cortex, medulla, renal pelvis, or any other related structures. The other internal organs were grossly normal, and no evidence of duplication was observed. 

## Discussion

As previous studies showed monocephalus dipygus has been reported in domestic animals such as sheep^7^^-^^9^ goats^2^^,^^5^^,^^10^ and very rarely in horses^11^ dogs and cats.^12^^-^^15^ It is believed that the anomaly is more common in cattle and usually affects the anterior part of body.^1^^,^^6^^,^^9^^,^^16^^-^^18^ The basic causes and mechanisms of caudal duplication and congenital limb deformities are still not well understood.

Beside genetic alterations, environmental factors such as intake of lupinus species, viral infections or exogenous hormone treatments are suspected to cause congenital duplications. Another hypothesis considers the ovulation of over-aged oocytes as a possible trigger.^6^

Conjoined twins classified as free or attached symmetrical or free or attached asymmetrical.^5^ Shojaei *et al*. reported a monocephalus dipygus female Holstein calf in Kerman, south of Iran but there was some difference between that case and the present report; in that case two small supernumerary medial limbs were observed, so the case was classified as conjoined symmetrical twin, also the urogenital system was affected and two uterine and urinary bladder were observed.^1^ The present case showed a monocephalus dipygus male Holstein calf with an extra pelvic limb attached to the uncompleted pelvic in one side and just a short bony structure in other side. Therefore, classification has been made as asymmetrical attached twins; the genital system was not affected and just one extra kidney- like structure was found. 
